# Integrating humanities in healthcare: a mixed-methods study for development and testing of a humanities curriculum for front-line health workers in Karachi, Pakistan

**DOI:** 10.1136/medhum-2022-012576

**Published:** 2024-01-17

**Authors:** Danya Arif Siddiqi, Fatima Miraj, Mehr Munir, Nowshaba Naz, Asna Fatima Shaikh, Areeba Wajahat Khan, Shama Dossa, Inamullah Nadeem, Monica J Hargraves, Jennifer Urban, Mubarak Taighoon Shah, Subhash Chandir

**Affiliations:** 1 IRD Global, Singapore; 2 IRD Pakistan, Karachi, Pakistan; 3 Habib University, Karachi, Pakistan; 4 Hargraves Consulting, Little Rock, Arkansas, USA; 5 Montclair State University, Montclair, New Jersey, USA

**Keywords:** Literature, Medical humanities, Primary care, literature and medicine

## Abstract

Lady health workers (LHWs) provide lifesaving maternal and child health services to >60% of Pakistan’s population but are poorly compensated and overburdened. Moreover, LHWs’ training does not incorporate efforts to nurture attributes necessary for equitable and holistic healthcare delivery. We developed an interdisciplinary humanities curriculum, deriving its strengths from local art and literature, to enhance character virtues such as empathy and connection, interpersonal communication skills, compassion and purpose among LHWs. We tested the curriculum’s feasibility and impact to enhance character strengths among LHWs.

We conducted a multiphase mixed-methods pilot study in two towns of Karachi, Pakistan. We delivered the humanities curriculum to 48 LHWs via 12 weekly sessions, from 15 June to 2 September 2021. We developed a multiconstruct character strength survey that was administered preintervention and postintervention to assess the impact of the training. In-depth interviews were conducted with a subset of randomly selected participating LHWs.

Of 48 participants, 47 (98%) completed the training, and 34 (71%) attended all 12 sessions. Scores for all outcomes increased between baseline and endline, with highest increase (10.0 points, 95% CI 2.91 to 17.02; p=0.006) observed for empathy/connection. LHWs provided positive feedback on the training and its impact in terms of improving their confidence, empathy/connection and ability to communicate with clients. Participants also rated the sessions highly in terms of the content’s usefulness (mean: 9.7/10; SD: 0.16), the success of the sessions (mean: 9.7/10; SD: 0.17) and overall satisfaction (mean: 8.2/10; SD: 3.3).

A humanities-based training for front-line health workers is a feasible intervention with demonstrated impact of nurturing key character strengths, notably empathy/connection and interpersonal communication. Evidence from this study highlights the value of a humanities-based training, grounded in local literature and cultural values, that can ultimately translate to improved well-being of LHWs thus contributing to better health outcomes among the populations they serve.

## Introduction

Front-line health workers (FHWs) (also referred to as community health workers, community health representatives, public health aid, lay health advisors), (Love, Gardner, and Legion 1997) are at the core of realising health coverage goals by facilitating access to and utilisation of health services, especially in low-income and middle-income countries (LMICs). Working with poor health infrastructure, high disease burden and dismal health indicators, FHWs are trusted members of society, who strengthen the public healthcare system by increasing outreach for primary preventive and curative health services to underserved populations (APHA 2014). FHW programmes are common across LMICs. For example, India’s Accredited Social Health Activists (Sharma, Webster, and Bhattacharyya 2014) the Shasthya Shebikas in Bangladesh, (Joardar *et al*. 2020) the Health Development Army Volunteers of Ethiopia, (Maes *et al*. 2018) and Kaders in Indonesia, (Perry 2020) all work towards a common goal to promote primary healthcare in their respective countries, especially for women and children. However, they are often faced with harsh working conditions.

In Pakistan, FHWs consist of lady health workers (LHWs), community health workers, vaccinators and midwives, primarily appointed by the government to address the country’s high maternal and child morbidity and mortality rates. The LHW Programme was launched in Pakistan in 1994 with the aim of increasing utilisation of health services at the community level. There are currently 125 000 women employed in the LHW programme, making it one of the largest front-line worker programmes in the world. LHWs have been at the forefront of improving Pakistan’s primary healthcare. For almost 60% of the population, especially women and children, LHWs are the first and only point of contact with the health system (Haq, Iqbal, and Rahman 2008). LHWs’ years of concerted efforts have significantly contributed to improved maternal and child health outcomes in Pakistan. They are also at the front line of polio eradication efforts in the country. LHWs’ role became more prominent in response to unprecedented challenges of face-to-face health delivery brought about by the COVID-19 pandemic.

Despite the centrality of their role, LHWs do not receive adequate non-technical training and support (APHA 2014). LHWs are trained on the more technical aspects of their jobs, consisting of 3 months of classroom-based training and 12 months of rigorous on-the-job training. After this, LHWs receive ongoing refresher trainings throughout their tenure, typically 1 day of training per month organised at their primary health centre (Lassi, Kahn, and Zulliger 2020). They frequently report dissatisfaction, occupational stress and security concerns, and suffer work burn-out and fatigue (Jaskiewicz and Tulenko 2012). These heightened stress levels are triggered by poor and irregular financial compensation, archaic patriarchal norms and lack of rapport with new communities (Carr *et al*. 2021), leading to lower productivity and motivation, thus diminishing the inherent meaningfulness of their work (Carr *et al*. 2021; Mumtaz *et al*. 2003). Incidents of violence against LHWs are frequent since they operate in inaccessible and dangerous areas (Shaikh *et al*. 2020). Notably, LHWs have been subjected to attacks, often facing death during the polio campaigns (DAWN 2020). The onset of COVID-19 compounded the challenges faced by LHWs, negatively impacting their productivity and motivation, weakening their resolve and self-worth, and eventually limiting their ability to provide high-quality healthcare (Qasim *et al*. 2020). In concert with attempts at systemic reforms, it is imperative to remodel LHWs’ current technical training to encourage empathic client understanding and help LHWs persevere through their working conditions. The research literature on empathy suggests that increasing empathy can contribute to improved connections with others (Decety and Fotopoulou 2014), improved personal resilience (Stanley and Bhuvaneshwari 2018) and protecting oneself against burn-out and fatigue (Thirioux, Birault, and Jaafari 2016). Thus, our intervention was aimed at strengthening empathy, compassion and connection as an indirect means of reducing LHW stress, burn-out and lack of connection.

A renaissance of professional training is occurring globally with a shift towards a different paradigm of humanities-based curricula. Humanities entails the study and understanding of the human experience (behaviour, economies, culture and society) from a critical perspective (Wierzbicka 2011). In the literature on character development, the humanities is increasingly being recognised as an effective tool for nurturing character strengths and developing value literacy (Mar, Oatley, and Peterson 2009; Vitz 1990). In fact, the link between medicine and the humanities has existed since antiquity with some of the most prominent physicians of premodernity (Aristotle, Hippocrates, Maimonides and Chekov, among others) straddling the fields of medicine and the arts and humanities, and establishing medicine as part of intellectual and cultural life (Wald, McFarland, and Markovina 2019). While commenting on the importance of character education for solving real-world challenges, several studies have pointed out the inadequacy of technical training, and suggested integration of the humanities with professional education curricula (Ben-Haim 2000; Graham *et al*. 2016). Within the life sciences literature, studies have confirmed the feasibility and efficacy of deploying humanities curricula within medical education to increase empathy and professional satisfaction among medical practitioners (Graham *et al*. 2016; Shapiro, Morrison, and Boker 2004). Literature provides evidence for the potency of humanities-based curricula to enhance character strengths in multiple high-stress jobs and professions, and among college students (Carr *et al*. 2021; Kidd *et al*. 2016; Ramai and Goldin 2013). Humanities-based interventions have already been shown to increase trainees’ character strength virtues. Several studies have found that participating in humanities-based interventions increases empathy among healthcare professionals. For example, an empathy-based training programme in Turkey (Kahriman *et al*. 2016) resulted in improved empathetic behaviour among healthcare professionals. Nurses who participated in a humanities-based in-service training programme demonstrated increased empathy (Ançel 2006). In another study, a cohort of otolaryngology residents was given three 90 min sessions of empathy training, after which they reported feeling more empathy towards their patients (Riess *et al*. 2011). Literature has also shown that healthcare practitioners who score high on empathy and related virtues are more likely to achieve patient satisfaction, treatment compliance and health equity (Hojat *et al*. 2011; Riess 2015; Scheepers *et al*. 2015).

Research has also explored the pathways by which increased empathy is achieved through exposure to the humanities, which include an improved theory of mind (Kidd and Castano 2013) and neural responses in the brain (Zak 2015). The neuroscientific literature, although limited, sheds light on the pathways through which humanities can affect key character strengths, most notably empathy. Emerging research suggests that empathy is actually a learnable, ‘neurobiologically based competency’ (Riess 2017). Functional MRI studies have shown that empathy-related training and practices such as loving-kindness meditation not only improve scores of self-reported empathy but also promote activation of relevant brain structures (Shalev and McCann 2020). Similarly, the effects of fiction reading on empathy have been well studied. Fiction-reading and mentalisation are interrelated and activate the medial prefrontal cortex, bilateral posterior superior temporal sulcus and the anterior temporal regions of the brain that are also part of the default mode network that control mentalisation (the ability to understand mental states of self and others (Shalev and McCann 2020; Tamir *et al*. 2016)). Studies have also shown training-related changes in the neural responses to suffering (ie, greater compassion has been observed in groups that received the trainings) (Weng *et al*. 2013).

Despite abundant literature on the value of interdisciplinary and humanistic approaches to training, (Crawford *et al*. 2015) research demonstrating the impact of humanities on specific character strength outcomes, such as empathy and connection, compassion, purpose, interpersonal skills, joy, and self-worth is limited and ad hoc. Moreover, there is limited evidence regarding the feasibility and efficacy of implementing humanities curricula for front-line primary health workers (as opposed to physicians and nurses), who serve as an essential conduit between health systems and vulnerable populations in low-resource settings.

**Figure 1:**
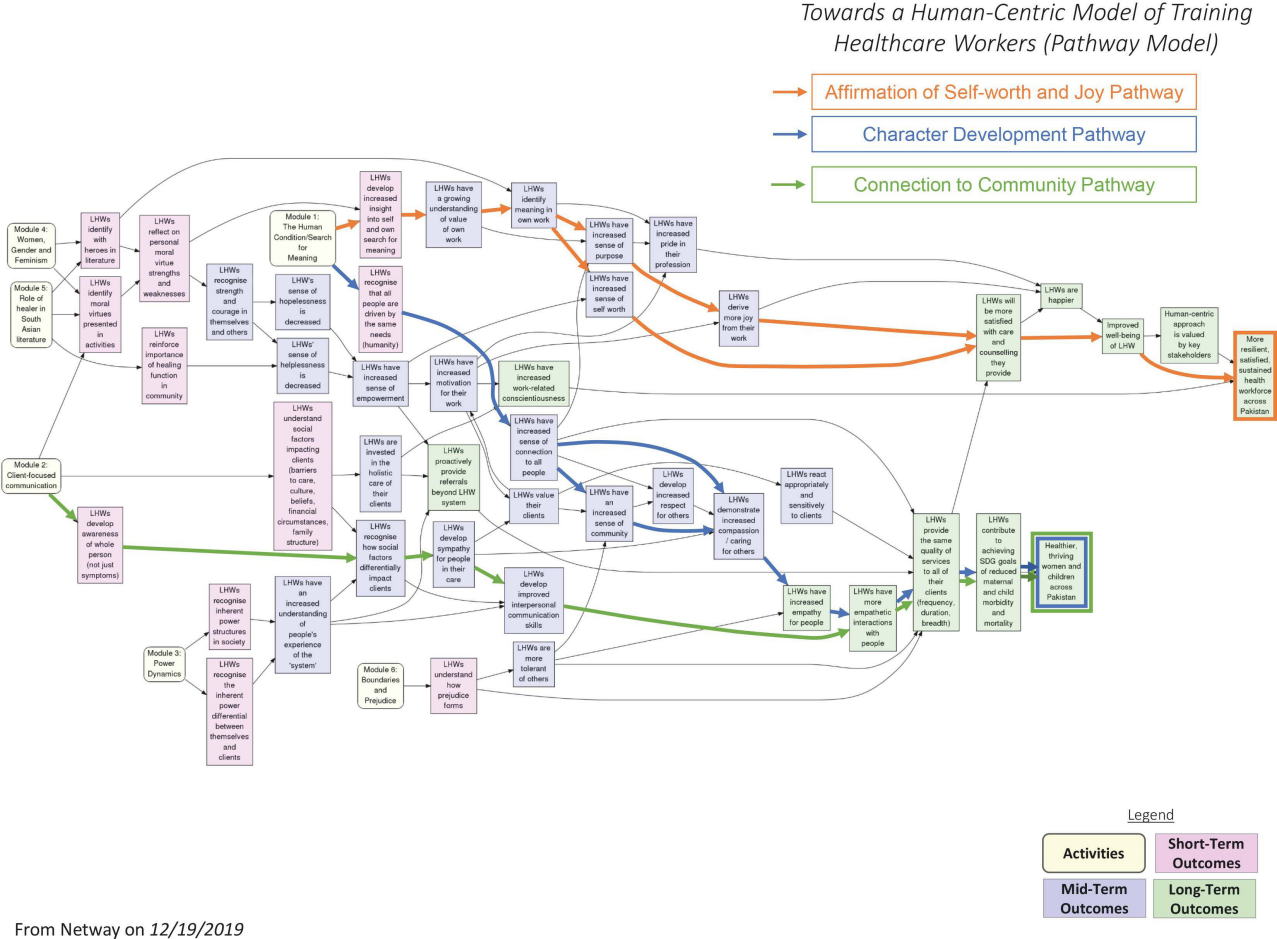
Pathway model. LHWs, lady health workers.

Our study was designed to embed the constructs of compassion, purpose and joy into one of the most important levels of healthcare delivery, and ultimately to transform the lives of both the providers and recipients through those constructs. Leveraging tools offered by the arts and humanities, our intervention aimed to hone healthcare workers’ ability to provide empathetic, person-centred care, and thus precipitate improvements in the health outcomes of the populations they serve. Simultaneously, the intervention also encouraged insight and self-reflection among participants, leading them to affirm and celebrate the value and meaningfulness of their own work. The intervention was designed to bring about change in three broad ways: by increasing LHWs’ sense of connection to all members of the communities they serve; by strengthening important character virtues of compassion and empathy; and by strengthening LHWs’ sense of self-worth and joy in their work. At the study outset, we used Relational Systems Evaluation to develop a pathway model which presents the programme’s underlying theory of change (Urban *et al*. 2021). The pathway model is presented in figure 1.

We developed an interdisciplinary humanities curriculum, deriving its strengths from local art and literature, to enhance character virtues such as empathy and connection, compassion, purpose, and to improve interpersonal communication skills among LHWs. We investigated the feasibility of implementing this local-language humanities curriculum among LHWs in a low-resource setting (Karachi, Pakistan) and evaluated the relationship between participation in the curriculum and the curriculum’s impact in building LHWs’ key character strengths.

## Methods

### Study design

We deployed a multiphase mixed-methods pilot study (including process and outcome evaluation) with an evolutionary evaluation approach, which stresses the importance of aligning evaluation methods with the life-cycle phase of the programme (Urban, Hargraves, and Trochim 2014). The two phases of the study included a development phase and an implementation phase. The quantitative component of the study involved administration of the Character Strength Survey with LHWs who received the intervention. The qualitative component comprised focus group discussions (FGDs) and cognitive interviews during the development phase to ensure contextual fit of the curriculum and relevance of the Character Strength Scale, respectively. In-depth interviews (IDIs) with participating LHWs (and lady health supervisors (LHS)) were conducted to gain insights into their experience of undergoing a humanities-based training.

#### Process evaluation

Since our intervention was being delivered for the first time, we conducted extensive process evaluation (during the development and implementation phases) in addition to assessing key programme outcomes. During curriculum development and refinement, we recorded feedback through FGDs and noted any text modifications. For character strength survey development and validation, we refined the survey through cognitive interviews, back-translations and pretesting. Data on process assessment for the curriculum delivery were generated through three methods. First, we collected participant feedback through short post-session feedback surveys. These required participants to rate the sessions, elaborate on what they liked and disliked about the session and their feedback on the facilitator’s teaching style. Second, we recorded facilitator’s feedback of participant engagement and overall performance collected through feedback forms completed by the facilitator after each session. Lastly, observer feedback was recorded by two study team members taking observation notes during the sessions that outlined their subjective understanding of the facilitator’s lectures, participants’ responses and the overall environment of the sessions. They also recorded process indicators such as attendance, degree of participant engagement and number of times participants initiated discussions.

#### Outcome evaluation

Our primary outcomes included empathy (ethnocultural and religious) and connection, compassion, purpose and interpersonal communication skills. Our secondary outcomes included sense of joy and self-worth. These were evaluated through the character strength survey and through the postintervention IDIs.

### Study site

We conducted the study in Bin Qasim (District Malir) and Korangi towns (District Korangi), in Karachi, Pakistan. Karachi is the largest metropolitan hub in Sindh province, with a diverse population of over 16 million people. These two towns were selected based on the demographic variability, varied levels of education and multiple ethnicities.

### Study population

We recruited 48 LHWs (sample size determined through convenience sampling) using age-stratified random sampling from a total of 368 LHWs deployed in Karachi (148 from Korangi and 220 from Bin Qasim) and 10 LHS who supervised the participating LHWs. To participate, LHWs had to be working in the selected towns, agree to attend all sessions and receive approval from their immediate supervisor.

### Patient and public involvement

Participating LHWs provided written informed consent.

### Study procedure and data collection

#### Development phase

##### Humanities curriculum

We developed an interdisciplinary humanities curriculum for LHWs in collaboration with faculty from the Social Development and Policy and Comparative Humanities Program at Habib University, Pakistan. The curriculum was developed in Urdu (local language) and consisted of local literary texts, dramas and poetry intertwined with interactive activities, adopting an andragogy that supported the education level and experiences of the LHWs. The curriculum was designed to: strengthen and expand LHWs’ sense of self-worth and experience of joy in their work; promote essential character virtues related to compassionate caregiving; and strengthen LHWs’ feelings of connection to community (particularly across differences due to ethnic or other boundaries). Further details on the content of the curriculum can be found in the online supplemental material.

10.1136/medhum-2022-012576.supp1 Supplementary data

We conducted four FGDs during the curriculum development stage with a total of 28 LHWs (who were not part of the randomised sample selected for training) and 6 field officers (organisation employed staff with more than 5 years of field experience implementing projects and interacting with FHWs and communities).

##### Character strength survey

We developed a multidimensional character strengths survey through contextual adaptation of previously validated tools (Hojat *et al*. 2018; Steger, Dik, and Duffy 2012; Wagaman *et al*. 2015; Wang *et al*. 2003), followed by pretesting and refinement of the study-tailored survey tool. The final survey can be found in the online supplemental material.

10.1136/medhum-2022-012576.supp2 Supplementary data

#### Implementation phase

We trained 48 LHWs on the curriculum which consisted of 12 modules spanning 12 weeks (15 June to 2 September 2021). The curriculum was delivered by one trained facilitator to 48 LHWs divided equally into three groups (A, B, C; 16 LHWs per group) to ensure adherence to the COVID-19 social distancing protocol. Sessions consisted of interactive discussions and activities (narrative writing, role-playing, singing, and viewing of short films and documentaries) based on carefully selected texts and other media such as music, poetry and film.

Each of the 36 sessions was designed to be approximately 3 hours long. Our training schedule was disrupted due to poor weather conditions caused by monsoon rain, which made it difficult for participants to reach the training venue, as well as due to COVID-19 lockdowns imposed between June and September 2021. Taking into consideration LHWs’ busy work schedule, we had to increase the duration of 11 sessions to 6 hours (with a 1 hour break in between) to complete our intervention on time.

The survey was administered to all participants at three time points (baseline, endline and a 4-month follow-up) to examine changes in four character constructs: empathy/connection, interpersonal communication skills, compassion and purpose.

We also conducted IDIs 3 months post-intervention with 12 randomly selected LHWs and 10 LHSs. IDIs were conducted to enrich our quantitative results with detailed qualitative feedback and enabled us to collect feedback on the training curriculum. The IDIs with LHS focused on understanding what change, if any, was observed by the supervisors in the LHWs post training. IDIs were conducted in person, by one project member accompanied by a notetaker. Interview duration was 50–60 min with LHWs and 30 min with LHS.

### Outcomes

Our outcome variables are defined below.

*Empathy towards and connection with communities*. Empathy is comprised of several subcomponents including: empathy for people belonging to cultures and ethnicities different from one’s own; a sense of connection to the community by transcending cultural boundaries; empathy for those prescribing to a religious faith different from one’s own (religious bias); and empathy toward healthcare clients.

To address empathy/connection to people belonging to cultures and ethnicities different from one’s own, we adapted 16 items from the Scale of Ethnocultural Empathy (SEE) (Wang *et al*. 2003). A sample item is ‘It is easy for me to understand what it would feel like to be a person of another ethnic background other than my own’. Responses were on a 5-point Likert-type scale ranging from 1=strongly disagree to 5=strongly agree. Sense of connection to the community by transcending cultural boundaries was also measured using SEE. A sample item is ‘I am aware of how society differentially treats ethnic groups other than my own’.

To measure religious bias we adapted nine items from the original SEE (Wang *et al*. 2003). A sample item is ‘It is easy for me to understand what it would feel like to be a person of another religious background other than my own’. Responses were on a 5-point Likert-type scale ranging from 1=strongly disagree to 5=strongly agree.

To measure empathy/connection towards healthcare clients we adapted 20 items from the Jefferson Scale of Empathy (JSE) (Hojat *et al*. 2018). A sample item is: ‘I try to understand what is going on in my clients minds by paying attention to their non-verbal cues and body language’. Responses were on a 7- point Likert-type scale ranging from 1=strongly disagree to 7=strongly agree.

*Compassion*. We measured LHWs’ compassion towards their clients, including their resolve for helping and being of service to others; having positive feelings about their ability to help others; and deriving pleasure and satisfaction from being able to do their work well. We adapted 10 items from the Compassion Satisfaction subscale of the 30-item Professional Quality of Life (ProQOL) Scale (Wagaman *et al*. 2015). A sample item is: ‘I get satisfaction from being able to help people’. Responses were on a 5-point Likert-type scale ranging from 1=never to 5=very often.

*Interpersonal communication skills*. To measure how LHWs put their interpersonal communication skills to use when conversing with their clients who may be ethnically, culturally or religiously different from their own we used 16 items from the SEE, 19 items from the JSE and 9 items from the scale of ethnocultural empathy-religious (SEE-R).

*Purpose*. To measure sense of meaningful work and a sense of purpose in life achieved through work we adapted 10 items from the Work and Meaning Inventory (WAMI) (Steger, Dik, and Duffy 2012). A sample item is ‘I understand how my work contributes to my life’s meaning’. Responses were on a 5-point Likert-type scale ranging from 1=absolutely untrue to 5=absolutely true. Higher score indicated higher sense of meaningful work.

#### Secondary outcomes

*Self-worth*. To measure valuing oneself as a person we used IDI responses to the question ‘Can you please describe any changes you felt in yourself since participation in the program?’.

*Joy*. We measured joy through IDIs with LHWs that took place post-intervention, where LHWs were asked to reflect and self-report the changes they felt withinecause of the intervention.

### Statistical analysis

#### Quantitative analysis

For descriptive analysis, we used frequencies (%) for categorical variables, and mean and SD for continuous variables. The baseline, endline and follow-up scores were compared for each of the outcomes along with the mean difference between baseline and endline, and baseline and follow-up. We used a paired t-test to detect whether the change in scores was statistically significant. We used subscale scores for constructing composite variables for further analysis, according to the guidelines of the original scale (Hojat *et al*. 2018; Steger, Dik, and Duffy 2012; Wang *et al*. 2003) and in line with the similar approach described elsewhere (Sullivan and Artino 2013). All quantitative analyses were performed using Stata, V.14.2 (StataCorp, College Station, Texas, USA).

To account for missing values in the JSE, if 80% of the items were attempted, the response was used, and the missing values were populated by the scale mean values; this method was prescribed by the original authors of the JSE. In the other scales, if 90% of the items were attempted, the responses were used. The missing values were populated by the mean values of the scales. Observations for participants who did not complete 80% items in the JSE or 90% of items in the other scales were dropped from the final analysis.

#### Qualitative analysis

Interviews were audio-recorded with consent from the participants. Interview recordings were transcribed in Urdu and translated into English for qualitative analysis. Qualitative data were analysed using NVIVO V.14. All transcripts were separately coded by two independent researchers. Both researchers compared their codes to reach a consensus and scrutinised the results to ensure trustworthiness and comprehensiveness of the analysis. Major themes were then extracted through thematic analysis.

## Results

### Participant characteristics

The sample consisted of 47 LHWs whose average age was 47.5 years (SD=7.3) (table 1). On average, participants had 10.3 (SD=1.9) years of education and 19.3 (SD=4.6) years of work experience. Most LHWs were married (68.1%; 32/47) while 17.0% (8/47) were widowed, 10.6% (5/47) divorced and 4.3% (2/47) were unmarried. Most of the LHW’s were the primary earners in their households (93.6%; 44/47). More than half (66.0%; 31/47) of the LHWs reported working for 4–8 hours a day, and 72.3% (34/47) reported that they visited 7–10 households per day.

**Table 1:** Demographic characteristics of lady health workers (LHWs) who participated in a humanities curriculum training administered from 15 June 2021 to 2 September 2021 (n=47)

	N	%
Age (years)		
31–35	2	4.3
36–40	7	14.9
41–45	11	23.4
46–50	7	14.9
51–55	13	27.7
56–60	7	14.9
Marital status		
Married	32	68.1
Unmarried	2	4.3
Widowed	8	17.0
Divorced	5	10.6
Number of family members		
1–3	5	10.6
4–6	29	61.7
7 or more	13	27.7
Monthly household income*		
<US$183	10	21.3
US$183–360	24	51.1
>US$367	13	27.7
Primary earner(s) in the family†		
Self	44	93.6
Husband	20	42.6
Sister	2	4.4
Son	5	10.6
Mother	1	2.1
Primary earner’s occupation†		
LHW	31	66.0
Private sector	10	21.3
Labourer (mazdoor, mechanic, plumber)	6	12.8
Driver	3	6.4
Other	5	10.6
Ethnicity		
Balochi	4	8.5
Punjabi	11	23.4
Sindhi	11	23.4
Urdu speaking	21	44.7
Spoken languages†		
Urdu	47	100.0
Sindhi	17	36.2
Punjabi	15	31.9
Balochi	4	8.5
Pashto	1	2.1
Other	2	4.3
Preferred language spoken at home†		
Urdu	29	60.4
Sindhi	11	22.9
Punjabi	9	18.7
Balochi	4	8.3
Cellphone access (yes)	47	100.0
Type of cellphone		
Smartphone	35	74.5
Keypad phone	12	25.5
Computer access (yes)	3	6.4
Internet access (yes)	22	46.8
Mode of transport to work†		
On foot	35	74.5
Bus	5	10.6
Rickshaw	10	21.3
Private vehicle	2	4.3
Types of training received†		
Family planning	34	72.3
Antenatal care	24	51.1
Nutrition	25	53.2
Vaccination	40	85.1
Deworming	21	44.7
Soft skills	25	53.2
Other	15	31.9
Types of immunisation campaigns LHW participated in†		
Measles	46	97.9
Polio	47	100.0
TCV	42	89.4
Other	11	23.4
Received sufficient training from LHW programme		
Yes	32	68.1
No	10	21.3
Not sure	5	10.6
Average daily working hours		
1–4 hours	12	25.5
4–8 hours	31	66.0
8 hours or more	4	8.5
Average number of houses visited per day		
5–7 houses	6	12.8
7–10 houses	34	72.3
10 houses or more	7	14.9

	Mean	SD
Age (years)	47.5	7.3
Education (years)	10.3	1.9
Work experience (years)	19.3	4.6
Monthly household income (US$)*	297.8	127.1

*Conversion rate: 1 US$=PKR163.7.

†Multiple responses allowed.

PKR, Pakistani Rupee; TCV, typhoid conjugate vaccine.

### Process evaluation results

In total, 47 of the 48 (98%) LHWs completed the training, 1 dropped out due to personal reasons. 34 (71%) LHWs attended all 12 sessions and maintained 100% attendance. On average, one LHW participated 13.4 times during a session (range: 2.8–32.2), with LHWs asking questions or initiating new discussions on their own on average 0.7 times per session (range: 0–1.8). Evaluation results from the participant, facilitator and observer feedback are reported in the online supplemental section.

10.1136/medhum-2022-012576.supp3 Supplementary data

### Outcome evaluation results

Table 2 shows the baseline, endline and 4-month follow-up mean scores for the survey, arranged by the different constructs. It demonstrates the comparison of mean scores between baseline and endline, baseline and follow-up, and endline and follow-up. The latter two scores help measure the retention of the curriculum over time.

**Table 2:** Comparison of character strength survey scores of 47 lady health workers (LHWs) administered between Jun 15, and Jan 21, 2022, before and after attending a humanities training

										Baseline-Endline				Baseline-Follow-up				Endline-Follow-up			
Scale	Baseline Scores			Endline Scores			4 Month Follow-Up Scores			Mean Difference	95% CI		P value	Mean Difference	95% CI		P value	Mean Difference	95% CI		P value
	n	Mean	SD	n	Mean	SD	n	Mean	SD												
Empathy towards and connection with communities and Interpersonal Communication Skills																					
Scale of Ethnocultural Empathy (80)	45	51.8	7.3	45	56.5	8.7	43	56.8	9.6	4.7†	(1.35)	(8.11)	0.007	5.0†	(1.43)	(8.68)	0.007	0.3	(−3.58)	(4.18)	0.878
Empathetic Perspective Taking (35)		22.4	3.7		24.5	4.2		24.0	4.2	2.1*	(0.44)	(3.78)	0.014	1.6	(−0.10)	(3.26)	0.655	−0.5	(−2.28)	(1.28)	0.578
Acceptance of Cultural Differences (25)		15.7	5.0		17.8	5.4		17.0	5.9	2.1	(−0.09)	(4.27)	0.060	1.3	(−0.99)	(3.66)	0.257	−0.8	(−3.20)	(1.60)	0.508
Empathetic Awareness (20)		13.6	4.2		14.2	4.2		15.8	3.7	0.5	(−1.23)	(2.30)	0.549	2.2*	(0.47)	(3.83)	0.013	1.6	(−0.08)	(3.28)	0.062
Scale of Ethnocultural Empathy -Religion (60)	45	37.8	4.8	45	41.3	7.3	43	40.5	7.1	3.4*	(0.84)	(6.02)	0.010	2.7*	(0.12)	(5.23)	0.040	−0.8	(−3.85)	(2.25)	0.604
Jefferson Scale of Empathy – HP (140)	45	95.1	18.3	45	105.0	15.2	43	103	18.4	10.0†	(2.91)	(17.02)	0.006	8.0*	(0.17)	(15.77)	0.045	−2.0	(−9.14)	(5.14)	0.579
Compassion																					
Professional Quality of Life - Compassion Satisfaction subscale (50)	46	47.3	4.5	46	48.1	2.3	44	47.6	3.4	0.8	(−0.72)	(2.26)	0.309	0.3	(−1.42)	(1.96)	0.751	−0.5	(−1.71)	(0.71)	0.414
Purpose																					
Work and Meaning Inventory (50)	44	45.3	3.4	44	46.1	3.1	42	45.6	3.5	0.9	(−0.51)	(2.24)	0.215	0.3	(−1.09)	(1.83)	0.617	−0.5	(−1.92)	(0.92)	0.484
Positive Meaning (20)		18.6	1.7		18.8	1.7		18.9	1.7	0.2	(−0.50)	(0.95)	0.535	0.3	(−0.42)	(1.05)	0.397	0.1	(−0.63)	(0.83)	0.786
Meaning-Making from Work (15)		14.2	1.1		14.5	1.0		14.2	0.9	0.2	(−0.24)	(0.65)	0.36	0.0	(−0.47)	(0.41)	0.876	−0.3	(−0.71)	(0.11)	0.148
Greater Good Motivations (15)		12.4	2.2		12.8	1.8		12.5	1.8	0.4	(−0.43)	(1.29)	0.32	0.1	(−0.77)	(0.95)	0.837	−0.3	(−1.07)	(0.47)	0.442

*p < 0.05.

†p < 0.01.

#### Empathy/connection and interpersonal communication skills

We observed an increase in empathy and connection and interpersonal communication skills as measured by the SEE, SEE-R and JSE Scales. A significant increase in overall SEE mean scores was observed between baseline and endline (4.7 (51.8 vs 56.5; 95% CI 1.35 to 8.11, p=0.007)) and baseline and follow-up (5.0 (51.8 vs 56.8; 95% CI 1.43 to 8.68, p=0.007)). The difference in overall SEE scores between endline and follow-up (0.3 (56.5 vs 56.8; 95% CI −3.58 to 4.18, p=0.878)) was insignificant.

We observed a significant increase in overall SEE-R mean scores between baseline and endline (3.4 (37.8 vs 41.3; 95% CI 0.84 to 6.02, p=0.010)) and baseline and follow-up (2.7 (37.8 vs 40.5; 95% CI 0.12 to 5.23, p=0.040)). A decrease in scores was observed between endline and follow-up (−0.8 (41.3 vs 40.5; 95% CI −3.85 to 2.25, p=0.604)), indicating declining religious empathy over time since the training.

Scores on the JSE showed a 10 percentage point increase between baseline and endline (10.0 (95.1 vs 105.0; 95% CI 2.91 to 17.02, p=0.006)) and an 8.0 percentage point increase between baseline and follow-up (8.0 (95.1 vs 103.0; 95% CI 0.17 to 15.77, p=0.045)). A decrease in scores was observed between endline and follow-up (−2.0 (105.0 vs 103.0; 95% CI −9.14 to 5.14, p=0.579)) however, indicating declining empathy over time.

#### Compassion

Based on scores of the ProQOL Scale, we did not observe a significant increase in compassion post-survey, primarily because baseline scores were already high indicating potential ceiling effects. There was an absolute increase from baseline to endline (0.8, 95% CI −0.72 to 2.26, p=0.309) which dropped to an absolute increase of 0.3 points (95% CI −1.42 to 1.96, p=0.751) from baseline to follow-up.

#### Purpose

We observed an increase in the overall mean scores between baseline and endline (0.9 (45.3 vs 46.1; 95% CI −0.51 to 2.24, p=0.215)) and baseline and follow-up (0.3 (45.3 vs 45.6; 95% CI −1.09 to 1.83, p=0.617)) and a decrease in scores between endline and follow-up (−0.5 (46.1 vs 45.6; 95% CI −1.92 to 0.92, p=0.484)). However, none of these showed a significant difference, primarily because baseline scores were already high.

Overall, we saw significant positive increases in scores for empathy which declined over time since the training. No significant increases were observed for sense of purpose and compassion, due to ceiling effects.

### IDIs with LHWs

#### Feedback and experience

All 12 LHWs who were interviewed provided positive feedback on the training.1 All of them appreciated the sessions’ comfortable and friendly environment and mentioned that it allowed them to share their thoughts freely. Bushra commented:

*The environment was very friendly, if someone didn’t understand a question [facilitator] would answer it, no one would criticize them. (IDI-6*)

All LHWs praised the facilitator’s friendly and interactive teaching style, along with her ability to make everyone feel included and respected. Saira remarked:

*[The facilitator] would explain every little thing in great detail, such as why or how something happened [in the content]. Her teaching style was great. (IDI-12*)

In terms of the curriculum being taught, the majority of LHWs (75%; 11/12) gave positive feedback about the content, specifying that it had reminded them of things they had long forgotten (42%; 5/12). Many LHWs mentioned how the stories, riddles and poems discussed during the sessions would take them back to their childhood days and made them reminisce about happy times. Zara mentioned how one of the songs played in the sessions brought back memories of singing with her friends. She explained that despite having known about the song since her childhood, she only truly understood its meaning during the training. She also shared her experience of listening to the same song with her family after the training and interpreting its meaning for them.

Some LHWs (17%; 2/12) noted that the language in the content was difficult, however, one of them pointed out that she was able to understand it with the help of the facilitator. They also found the curriculum to be different from others they had been taught, and it allowed them to learn things beyond their technical work. As Saira mentioned,

*We would get handouts of the content which would include poetry, riddles, and stories. This training was quite different and very good.* (IDI-12)

All LHWs also listed different topics, essays or poems that they had enjoyed the most, and recalled how the examples given to them made them reflect on their own lives and actions.

A few LHWs mentioned limitations of the training. These were related to the long-time duration (>3 hours) of the sessions (42%; 5/12), and use of difficult Urdu (local language) words (17%; 2/12). Some LHWs, despite knowing Urdu, predominantly worked with Sindhi populations, and were hence more fluent in the Sindhi language. All LHWs looked forward to participating in a similar training in the future and said they would recommend the training to their fellow LHWs.

#### Impact of the training programme

Almost all LHWs (92%; 11/12) found the training relevant and applicable to their role as FHWs. 83% (10/12) said they were able to apply the learnings in their practical life, especially in terms of changing their thoughts, behaviour and attitudes. More specifically, LHWs reported being happier and more patient, confident, empathetic, and positive after the training. Hina noted,

*A lot [has changed], we feel younger, and we are now happier while doing our work, we have become more passionate and active. (IDI-2*)

They also reported to have learnt how to take better care of themselves. Nabiha said,

*This training has given us the strength to move forward, to think about ourselves, to take out time for ourselves. (IDI-7*)

Moreover, LHWs (75%; 9/12) felt they were better able to communicate effectively in their work and personal lives because of what they were taught. They mentioned their relationship with their families and communities had improved. The training helped LHWs understand and connect better with their communities, as noted by Sabiha,

*I utilized the benefits of our training in my community. I started using easier language. If I am able to understand them, then they should (also) be able to understand what I am saying. (IDI-3*)

Haleema, realising the impact of their words and actions on others said,

*we should act in a way that others don’t get hurt because of us. (IDI-1*)

#### Suggestions/areas for improvement

The main suggestions put forth by the LHWs included having interactive and innovative activities within the trainings (25%; 3/12), having similar trainings more consistently (25%; 3/12), conducting trainings in LHWs’ offices instead of an external location (17%; 2/12) and using easier language in the content of the curriculum (8%; 1/12). One LHW, Saira, specifically mentioned desiring recurrent trainings, otherwise they would eventually forget what they were taught.

### IDIs with LHSs

All LHSs were aware of the humanities training programme that LHWs working with them underwent, as the LHWs had shared their stories and experiences with their fellow workers and supervisors. The LHSs felt this training was relevant to LHWs’ work and similar trainings should be held for others (LHWs and LHSs) as well. All LHSs believed that the training had positively impacted the LHWs, especially in improving their communication skills (70%; 7/10), and boosting their confidence, motivation and self-worth (70%; 7/10).

Farah noted a shift in LHWs communication skills,

*LHWs have become very interactive with the community and started liking their work more. I personally see a big change in their communication. (IDI-23*)

Sidra also talked about how the LHWs have become more confident and started enjoying their work. She further highlighted the training’s impact on LHWs’ work by sharing an experience of how LHWs now performed better in their field:

*I went with them for the measles campaign and saw [name] how LHWs were able to convince those people who were refusing. (IDI-16*)

The majority of LHSs (70%; 7/10) also felt that LHWs who were provided with the training exhibited a change in behaviour and attitude as compared with those who had not undergone such training. LHSs reported that after the training, LHWs had become more confident, started enjoying their work and communicated more effectively in the field.

## Discussion

Our results indicate feasibility of deploying an innovative, interdisciplinary humanities curriculum, for nurturing key character strengths such as empathy and connection, interpersonal communication skills, compassion, and purpose among LHWs. Notably, the humanities-based training resulted in a significant 10-point increase in the pre-post scores for empathy/connection. Our findings provide evidence that a humanities curriculum can supplement LHWs’ traditional training to improve their well-being.

Participation in the humanities curriculum was associated with an improvement in all character strength scores from baseline to endline, with significant positive differences observed for empathy, especially for religious outgroups, interpersonal communication skills and connection to the communities. Evidence spanning several disciplines, including neuroscience, medicine and the humanities, has established a firm causal link between sustained engagement with humanities disciplines and higher empathy (Kidd and Castano 2013; Colvin *et al*. 2018; Halperin 2010). The increase in empathy brought about through sustained exposure to the humanities is a result of multiple pathways, including not only a deeper capacity to apprehend subjective realities, but also neural changes in the brain (Zak 2015). High empathy is in turn linked to better and more equitable health outcomes, and high patient satisfaction (Flickinger *et al*. 2016; Hojat *et al*. 2011; Squier 1990). Furthermore, providers with high empathy are themselves likely to experience more personal well-being, as they are more cognisant of the meaningfulness and human value of their work (Shanafelt *et al*. 2005; Thomas *et al*. 2007). Since part of the task of Medical Humanities is to refocus the gaze of the provider on the person receiving care, rather than the body that requires fixing, it further reinforces the sense of well-being, meaningfulness and human connection. Moreover, pedagogical techniques employed in humanities disciplines encourage critical thinking and creative reflection. This enhances creativity and agency, which are correlated with well-being.

Despite the positive findings, we observed a slight drop in scores from endline to follow-up, however, the follow-up scores were still higher than baseline scores. This suggests a need for refresher or periodic trainings to ensure knowledge is retained by the participants. Our findings are consistent with previous literature demonstrating increase in empathy, pre-intervention and post-intervention (Chapman *et al*. 2018) among nurses who were trained on empathy, communication, meditation and cultural competence (Bas-Sarmiento *et al*. 2019). In this study the empathy scores were similar immediately after the training and during a 1-month follow-up. An explanation for this difference from our finding (of lower follow-up scores) could be that our follow-up survey was conducted much later (after 4 months), implying a need for refresher training.

We also explored a dimension of empathy related to multiple religious denominations that coexist in Pakistan. Although the mean score for empathy towards multiple religious denominations was the lowest at baseline, a significant increase was observed post-intervention, supporting the positive impact a humanities training can have in fostering religious empathy in the local healthcare workforce. This holds immense importance in the Pakistani context, where a person’s religious and ethnic backgrounds shape the kinds of interactions people have with the healthcare system (Shoukat, Jafar, and Anjum 2022).

Studies measuring other constructs such as compassion and purpose corroborate our findings of improved outcomes following a humanities-based training (Bayer *et al*. 2021; Potter *et al*. 2013). For instance, Potter *et al*’s study evaluating compassion fatigue resiliency used the ProQOL Scale to establish the benefits of a compassion fatigue intervention for nurses (Potter *et al*. 2013). Similarly, Bayer *et al* used scores from the WAMI Scale to provide recommendations for interventions to foster meaning in work for healthcare workers (Bayer *et al*. 2021).

At a more personal level, LHWs found the humanities curriculum to be beneficial to their professional and personal lives, but also thoroughly enjoyed the innovative and novel content and delivery method. Participants reported improved confidence, interpersonal communication, and better understanding of self and their communities. Furthermore, a unique aspect of the humanities curriculum was its contextualisation in local cultural values and norms. Local language (Urdu) texts helped initiate discussion about pertinent social issues in the country and were able to successfully engage and influence participants.

To take our work forward, we propose a more rigorous evaluation of the humanities curriculum on selected character strength outcomes, along with expanding the curriculum to include other cadres of FHWs including vaccinators, midwives and community health workers. This would entail modifying the curriculum as well as the survey instruments to reflect the needs of the additional healthcare providers. We also propose to address ceiling effects in our survey tool (especially for the compassion measure) by collecting qualitative data that allow for more defined differentiation, as well as exploring other evaluation designs that allow us to compare our outcomes with healthcare providers who did not receive the humanities-based training. A key policy implication of our work is also the need for regular humanities-oriented refresher trainings for FHWs, as part of their continuous professional development.

### Limitations

Since this was a pilot for feasibility and acceptability of a humanities curriculum for health workers, our sample size was small (n=48) restricting our generalisability. Furthermore, due to the COVID-19 pandemic, we experienced some unscheduled delays in our intervention and were forced to overcome these by conducting longer training sessions. Such gaps and delays in training may have affected the participants’ learning and knowledge retention, and longer sessions might have impacted LHWs’ concentration and interest due to fatigue.

## Conclusion

A humanities-based training leveraging local arts and literature is a feasible intervention that demonstrates promise for nurturing key character strengths among LHWs. Evidence generated from this study highlights the importance of innovative training, grounded in local literature and cultural values, that goes beyond technical and theoretical skills and seeks to develop core character strengths among front-line workers. Training that focuses on character development can enable front-line workers to effectively address some of the most pressing issues such as polio eradication and is even more relevant in COVID-19 times when the healthcare workforce is recovering from the psychological aftermath of the pandemic. Further research is warranted to study the impact of the humanities curriculum among other cadres of front-line workers including vaccinators, community health workers and midwives, as well as its impact on long-term health outcomes of populations.

## Data Availability

Data are available upon reasonable request.

## Notes

1. All names are pseudonyms.
