# Supplementary material for: Integrating humanities in healthcare: a mixed-methods study for development and testing of a humanities curriculum for front-line health workers in Karachi, Pakistan
Source: Med Humanit. 2024 Jan 17;50(2):372–82. doi: 10.1136/medhum-2022-012576 (PMC11347256; doi:10.1136/medhum-2022-012576)
Supplement: Supplementary data [file medhum-2022-012576supp001.pdf]

Supplementary Material

Humanities Curriculum

The humanities curriculum was developed in collaboration with academic experts from Habib University. The curriculum encompassed six themes with six corresponding modules regarding each of the intended outcomes. The first module was about the "Search for Meaning and the Human Condition" in which different literary texts, poems, and songs were utilized to discuss what it really means to live a good, respectful life, and how meaning can be found in one's life and work. Interactive discussions on essays such as "Search of a Good life" by M. Ibrahim Khan prompted the participants to reflect on the sources of happiness in their lives.

The second module was regarding "Person-centered Communication and Care" in which participants' interpersonal communication skills towards their clients were discussed and honed further by reinforcing the concept of understanding and values of empathy with clients through self-reflection. Fehmida Riaz's poem "The Rain" taught the participants about understanding and respecting everyone's opinion as everyone has a different perspective on interpreting things. Recognizing and learning to respect different opinions allows participants to become more empathetic and compassionate healthcare providers. Interactive activities such as roleplays on a dialogue between two friends (neighbours) were added to encourage participants to speak up and equip themselves with the skills to interact with their fellows as well as their clients.

The third module talked about "Power Structures and the Broader Social Context" in which participants recognized the adverse effects of power relations on the most vulnerable and the role the participants may inadvertently play in this process. Participants also explored strategies to address power differentials and counter discrimination in their own work. The short film Balak was included in this module to help participants self-reflect on their actions and realize how one can often be quick to judge leading to wrongly discriminating against people.

The fourth module, "Women, gender, and the LHW mission" allowed the participants to understand the implications of patriarchy and gender discrimination, the concept of women's empowerment and its importance in their own lives and work. The intention for the module was to enable participants to have greater empathy for their clients as well as for themselves. The story "Maa Jee" (Mother) by Qudrat Ullah Shahab highlighted the importance of mothers and the essential role they play in an individual's life. The story highlighted the simplicity of a woman, especially a mother, who devotes her life for her children and family.

The fifth module, "Role of the healer in South Asian literature", used local literature to demonstrate the importance of the role of LHWs in the context of their communities and their ability to contribute to its wellbeing. Th content enabled participants to gain further understanding on the importance of empathy with clients. The life of Dr. Ruth Pfau was discussed to highlight her struggle in the fight for eradication of leprosy in Pakistan and for participants to learn from her story of dedication towards her role as a healer. Mother Teresa's poem, Jesay bhi ho ker guzaro (Go through it anyway) was read to emphasize the importance of forgiveness and honesty, as well as the importance of doing good, a lesson essential for LHWs where irrespective of the client's attitude or behaviour, LHWs or any health provider should provide the best possible care . A few lines from this poem are:

Jo achahi tum nae aaj ki hai,

Aksar ye hoga kay loag kaal bhool jain gaye.

Jaisay bhi ho achachi karo.

"The good you do today,

people will often forget tomorrow.

Do good anyway.

The last module, "Boundaries and Prejudice" gave the participants an understanding of the concept of discrimination and prejudice as well as its impact on vulnerable clients. Faiz Ahmed Faiz's poem "Mere dard ko jo zuban mily (If My Suffering Found a Tongue)" talks about the expression of one's pain and suffering and indicates how oppressed members or communities are often overlooked, and even among the oppressed there is discrimination e.g. women often hurt other women, or one religious group fights with another one. Faiz says in his poem".

Meri zaat zarra-e-bay-nishaan

Mere dard ko jo zuban milay

Mujhey apna naam-o-nishaan miley

My pain is the voiceless song

My being is nameless, void of identity

If my pain gets a voice..

I get a name, an identity

The story of the "Step mother" by Bheesham Shani, teaches a lesson of humanity and love. It talks about how a mother's love isn't confined by the relation of blood only, and can't be categorized into real or step mother. Such stories and poems led participants to develop self-awareness of their internalized prejudices to shift practices towards care giving and non-discrimination.

Supplementary Table 1: Outline of the 12 week humanities curriculum training for Lady Health Workers

**Module 1**	**Theme: Search for meaning and the human condition**	**Outcomes**
Week I	1.Introduction of participants and details of the study<br>2. Essay: Search of a good life (M Ibrahim Khan)<br>3. Story: The Kite Maker<br>4. Poem: *Bhulah Kia Janu Maen Kon* (Bhulay Shah)<br>5.Poem: A Question (Fatimah Hassan)	Participants will be able to:<br>• comprehend the objectives/ purpose of the humanities curriculum training<br>• understand the structure of the curriculum and course pedagogy<br>• collaboratively set course norms and expectations<br>• introduced to literature which will initiate a conversation on developing values of care, empathy, explore self-identity and self-worth in relation to work and clients<br>• develop useful communication skills for client interaction<br>• explore the concept of empathy<br>engage in a self-reflective exploration of their self-worth and self-respect
Week II	1.Essay: Respecting Oneself (Ibn e Habib)<br>2.Poem: The Ant (Zahra Nigar)<br>3.Song: *Anokha Ladla Khelan ko Mangy Chand* (Asad Muhammad Khan)<br>4.Urdu Riddles	
**Module 2**	**Theme: Person-centered communication and care**	**Outcomes**
Week III	Prose<br>1. Light-hearted (Tariq Hanif)<br>2. *Jeet Main Har Ya Har maen Jeet* (Tariq Hanif)<br>3. Story: First Death (Surender Parkash)<br>4. Poem: The Rain (Fehmida Riyaz)	• Participants will self-reflect to build on and reinforce their understanding and values of empathy with clients<br>• Participants will be able to strengthen their communication skills with clients
Week IV	1. Dialogue between friends (role play)<br>2. Essay on how to start a conversation with new friends	
**Module 3**	**Theme: Power structures and the broader social context**	**Outcomes**
Week V	1. *Bichri Konj* (Afsana)<br>2. Poem: *Aik Zindagi or Mil jaye* (Azra Abbas)<br>3. Poem : *Hath khol diye jayen* (Azra Abbas)	• Participants will explore and recognize the adverse effects of power relations on those most vulnerable and the role they themselves may inadvertently play in this process<br>• Participants will explore strategies to address power differentials and counter discrimination in their own practice
Week VI	1. Essay: The biggest crime (Tariq Hanif)<br>2. Essay: The Hope (Tariq Hanif)<br>3. Ghazal: *Khayal o Khawab hoe haen Mohabaten kaisi* (Ubaid Ullah Aleem)	

		• Participants will further develop their own strategies to address power differentials and counter discrimination in their own work
**Module 4**	**Theme: Women, gender, and the LHW mission**	**Outcomes**
Week VII	1. Essay: Women's Status<br>2. Fiction: *Maa Jee* (Qudrat Ullah Shahab)<br>3. Poem: In the Name of Artist (Ahmad Faraz)	• Participants will begin to explore the implications of the structure of patriarchy and gender discrimination<br>• Participants will be introduced to the concept of women's empowerment and its importance in their own lives and work
Week VIII	1. Essay: Seven Princesses of Shah Latif (Zahida Hina)<br>2. Essay: Women's plight in cultural rituals (Atiya Daoud)<br>3. Poem: Imprisoned Princess (Fehmida Riyaz)	
**Module 5**	**Theme: Role of the healer in South Asian literature**	**Outcomes**
Week IX	1. Dr Ruth Pfau: A servant Fairy (Rao Muhammad Shahid Iqbal)<br>2. Poem: Whatsoever the life may be (Mother Triessa)<br>3. Ghazal: *Diyar e dil ki rat maen chiragh sa jala gaya* (Nasir Kazmi)	• Participants will deconstruct the role of the healer in our context and the importance of their role as LHWs in their communities and their ability to contribute to its wellbeing<br>• Participants will gain further depth of understanding on the importance of empathy with clients
Week X	1. Story: Last Leaf (O Henry)<br>2. Poem: *Aey prindo kisi sham urty hoey* (Zeeshan Sahil)<br>3. Ghazal: *Ku ba Ku Phail gae bat Shanasai ki* (Parvin Shakir)	
**Module 6**	**Theme: Boundaries and prejudice**	**Outcomes**
Week XI	1. Poem: *Gham na Kar Gham na Kar* (Faiz Ahmad Faiz)<br>2. Poem: *Mery dard ko jo Zaban mily* (Faiz Ahmad Faiz)<br>3. Poem: *Phool Murjha gae haen saary* (Faiz Ahmad Faiz)<br>4. Essay: Fine Art is an effective mean of social changes (Irshad Bukhari)	• Understanding the concept of discrimination and prejudice as well as its impact on vulnerable clients<br>• Develop self-awareness for internalised prejudice and discrimination in order to shift practices towards non-discriminating caregiving
Week XII	1. Fiction: Step Mother (Bhesham Shani)<br>2. Poem: *Be qarai si be qarari hai* (Jhon Elia)	
