# Supplementary material for: Integrating humanities in healthcare: a mixed-methods study for development and testing of a humanities curriculum for front-line health workers in Karachi, Pakistan
Source: Med Humanit. 2024 Jan 17;50(2):372–82. doi: 10.1136/medhum-2022-012576 (PMC11347256; doi:10.1136/medhum-2022-012576)
Supplement: Supplementary data [file medhum-2022-012576supp002.pdf]

Supplementary Material

Towards a Human-centric model of training in healthcare - Character Strength Scale

<u>Section A (Work and Meaning Inventory)</u>

Work can mean a lot of different things to different people. The following items ask about how you see the role of work in your own life. Please honestly indicate how true each statement is foryou and your work. Please score each statement between 1 and 5, where 1 means it's absolutelyuntrue, and 5 means it is absolutely true. Please see annex. 1 for further scoring instructions.

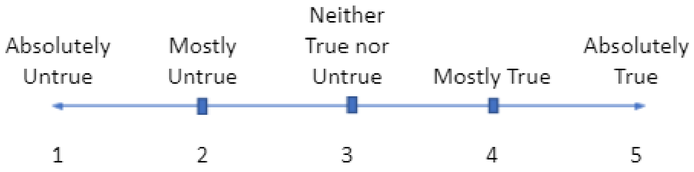

1. I have found a meaningful career.
2. I view my work as contributing to my personal growth.
3. My work really makes no difference to the world.
4. I understand how my work contributes to my life's meaning.
5. I have a good sense of what makes my job meaningful.
6. I know my work makes a positive difference in the world.
7. My work helps me better understand myself.
8. I have discovered work that has a satisfying purpose.
9. My work helps me make sense of the world around me.
10. The work I do serves a greater purpose.

Section B (Scale of Ethnocultural Empathy)

The next section is going to ask about how you feel about people who have a different ethnic background from you. Please score each statement between 1 and 5, where 1 means you strongly disagree with it, and 5 means you strongly agree. Please see annex. 2 for further scoring instructions.

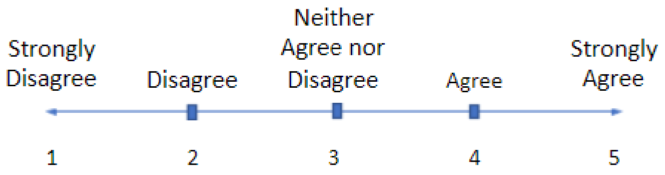

1. It is easy for me to understand what it would feel like to be a person of another ethnic background other than my own.
2. It is difficult for me to relate to stories in which people talk about ethnic discrimination they experience in their day to day lives.
3. It is difficult for me to put myself in the shoes of someone who is ethnically different from me.
4. I know what it feels like to be the only person of a certain ethnicity in a group of people.
5. I can relate to the frustration that some people feel about having fewer opportunities due to their ethnic backgrounds.
6. I feel uncomfortable when I am around a significant number of people who are ethnically different than me.
7. I don't know a lot of information about important social and political events of ethnic groups other than my own.
8. I feel irritated when people of different ethnic backgrounds speak their language around me.
9. I feel annoyed when people do not speak my language.
10. I get impatient when communicating with people from other ethnic backgrounds, regardless of how well they speak my language.

11. I do not understand why people want to keep their indigenous ethnic, cultural traditions instead of trying to fit into the mainstream.
12. I don't understand why people of different ethnic backgrounds enjoy wearing traditional clothing.
13. I am aware of how society differentially treats ethnic groups other than my own.
14. I recognize that the media often portrays people based on ethnic stereotypes.
15. I can see how other ethnic groups are systematically oppressed in our society.
16. I am aware of institutional barriers (e.g., restricted opportunities for job promotion) that discriminate against ethnic groups other than my own.

Section C (Professional Quality of Life – Compassion Satisfaction)

When you help people, you have direct contact with their lives. As you may have found, your compassion for those you help can affect you in positive and negative ways. This section will ask you about your motivation for helping people. Please score each statement between 1 and 5, where 1 means you never feel this way, and 5 means you feel it very often. Please see annex. 3 for further scoring instructions.

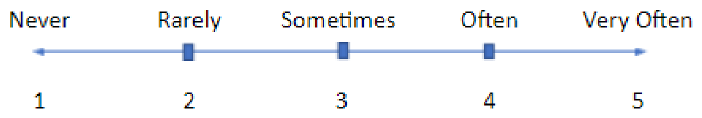

1. I get satisfaction from being able to help people.
2. I feel invigorated after working with those I help.
3. I like my work as a Lady Health Worker.
4. I am pleased with how I am able to keep up with helping techniques and protocols.
5. My work makes me feel satisfied.
6. I have happy thoughts and feelings about those I help and how I could help them.
7. I believe I can make a difference through my work.
8. I am proud of what I can do to help.
9. I have thoughts that I am a "success" as a Lady Health Worker.
10. I am happy that I chose to do this work.

Section D (Empathy towards other Religious Communities)

The next section is going to ask about how you feel about people who have a different religious background from yourself. Please score each statement between 1 and 5, where 1 means you strongly disagree with it, and 5 means you strongly agree. Please see annex. 4 for further scoring instructions.

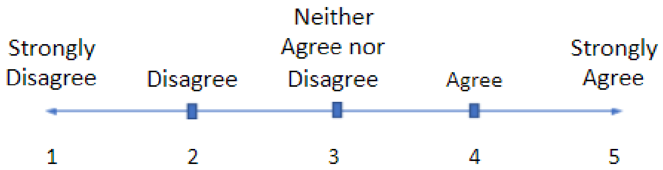

1. It is easy for me to understand what it would feel like to be a person of another religious background other than my own.
2. It is difficult for me to relate to stories in which people talk about religious discrimination they experience in their day to day lives.
3. I know what it feels like to be the only person of a certain religion in a group of people.
4. I can relate to the frustration that some people feel about having fewer opportunities due to their religious backgrounds.
5. I do not understand why people want to keep their religious-cultural traditions instead of trying to fit into the mainstream.
6. I am aware of how society differentially treats religious groups other than my own.
7. I recognize that the media often portrays people based on religious stereotypes.
8. I can see how other religious groups are systematically oppressed in our society.
9. I am aware of institutional barriers (e.g., restricted opportunities for job promotion) that discriminate against religious groups other than my own.

Section E (Jefferson Scale of Empathy – Health Professional version)

This section will ask you about how you provide treatment to your clients. Please score each statement between 1 and 7, where 1 means you strongly disagree with it, and 7 means you strongly agree. Please see annex. 5 for further scoring instructions.

.

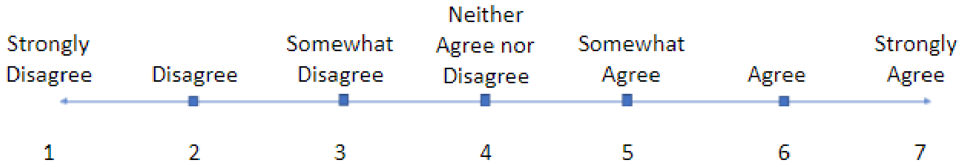

1. My understanding of how my clients and their families feel does not influence medical treatment.
2. My clients feel better when I understand their feelings.
3. It is difficult for me to view things from my clients' perspectives.
4. I consider understanding my clients' body language as important as verbal communication in caregiver-client relationships.
5. I have a good sense of humor that I think contributes to a better clinical outcome.
6. Because people are different, it is difficult for me to see things from my clients' perspectives.
7. I try not to pay attention to my clients' emotions in history taking or in asking about their physical health.
8. Attentiveness to my clients' personal experiences does not influence treatment outcomes.
9. I try to imagine myself in my clients' shoes when providing care to them.
10. My clients value my understanding of their feelings, which is therapeutic in its own right.
11. Clients' illnesses can be cured only by medical treatment; therefore, emotional ties to my clients do not have a significant influence on medical outcomes.
12. Asking clients about what is happening in their personal lives is not helpful in understanding their physical complaints.
13. I try to understand what is going on in my clients' minds by paying attention to their non-verbal cues and body language.
14. I believe that emotion has no place in the treatment of medical illness.
15. Empathy is a therapeutic skill without which success in treatment is limited.

16. An important component of the relationship with my clients is my understanding of their emotional status, as well as that of their families.
17. I try to think like my clients in order to render better care.
18. I do not allow myself to be influenced by strong personal bonds between my clients and their family members.
19. I do not enjoy reading non-medical literature or the arts.
20. I believe that empathy is an important therapeutic factor in medical treatment.

Appendix – Scoring Algorithms

A. Work And Meaning Inventory:
   i. This is a 10-item scale, which is further divided into 3 subscales:
      a) The "Positive Meaning" scale: add responses to items 1, 4, 5 and 8 for the subscale score (scored 4-20);
      b) the "Meaning-Making from Work" scale: add responses to items 2, 7 and 9 for the subscale score (scored 3-15);
      c) the "Greater Good Motivations" scale including items 3, 6 and 10 (scored 3-15). Subtract the rating for item 3 from 6 (e.g., if a respondent gave item 3 a rating of 2, then their converted rating would be 4 [6-2=4]);add this number to the ratings for items 6 and 10 to get the score.
   ii. The scores of these subscales are added together to get the test-taker's overall Meaningful Work score. **Higher score indicates higher sense of meaningful work**.
   iii. The respondent must answer 90% of the items to be considered a complete form. The remaining missing values are replaced with the mean values calculated from the items the respondent completed in the scale/subscale.

B. Scale of Ethnocultural Empathy - Ethnicity:

A five-point Likert Scale was used for the Scale of Ethnocultural Empathy (SEE). Despite trying to contact the author for several months, we were unable to get in touch and hence proceeded with the five-point scale used in most of the other scales in the instrument. However, after the pretesting activity, we recently heard back from the author and received the original scoring guidelines. For the final implementation of the SEE, we will therefore incorporate the original six-point scale, as shared by the author. The following algorithm and interpretation applies to both the five-point and six-point scales.

i. The scale is thematically divided into three sections: “Empathetic Perspective Taking”, “Acceptance of Cultural Differences”, and “Empathetic Awareness”

ii. For scoring, first reverse the score on items 2, 3, 6, 7, 8, 9, 10, 11, 12, then sum all scores. **Higher the scores, higher the level of ethnocultural empathy.**

iii. The respondent must answer 90% of the items to be considered a complete form. The remaining missing values are replaced with the mean values calculated from the items the respondent completed in the scale/subscale.

C. Professional Quality of Life Scale:

i. This is a 30-item scale, out of which we have selected 10 items of the Compassion Satisfaction subscale for our study. Higher scores on this subscale represent a greater satisfaction related to your ability to be an effective caregiver in your job.

ii. All respondents must select a number between 1-5 (1=never…5=very often). Their total score can range between 10-50.

iii. The original scale used “[*helper*]” to indicate someone who professionally helps or provides care for others. In our adaptation that was specified to Lady Health Worker. Items that used the iteration [*help*] were kept the same, but the brackets were removed.

iv. A score of 22 or less suggests a low compassion satisfaction level; between 23 and 41 suggests an average level of compassion satisfaction; and **42 or higher suggests a high level of compassion satisfaction of the respondent**.

v. The respondent must answer 90% of the items to be considered a complete form. The remaining missing values are replaced with the mean values calculated from the items the respondent completed in the scale/subscale.

D. Scale of Ethnocultural Empathy - Religion:

i. This scale was adapted by the study team from the original SEE scale, to measure the respondent's empathy towards other religious groups as well, since religion may be a dominant source of bias in Pakistani society. The items remained the same, except instead of "ethnicity" the term "religion" was used.

ii. The number of items was also reduced and only the ones applicable to religion were used in this scale.

iii. Similar to the SEE, scores of positively-worded items were added together using the standard method as in 1 (strongly disagree) to 5 (strongly agree). For scoring the negatively-worded or reversed items (2 and 5), subtract each score from 6, and add these scores to the rest.

iv. Adding the scores of all the items will give the cumulative scores for each subscale. We estimate that **the higher the score, the higher the level of empathy for people who have different religious beliefs** from those of the participants.

v. The respondent must answer 90% of the items to be considered a complete form. The remaining missing values are replaced with the mean values calculated from the items the respondent completed in the scale/subscale.

E. Jeffeson Scale of Empathy – Health Professional version:

i. This is a 20-item scale. Respondents must select a number between 1-7 (Strongly Disagree=1...Strongly Agree=7). Their score range may vary between 20-140.

ii. A respondent must answer at least 16 (80%) of the 20 items to be considered a complete form.

iii. If a respondent fails to answer 4 or fewer items, the missing values should be replaced with the mean score calculated from the items the respondent completed.

iv. Items 1, 3, 6, 7, 8, 11, 12, 14, 18, and 19 are reverse scored items (i.e., Strongly Agree=1...Strongly Disagree=7) while the other items are directly scored on their Likert weights (i.e., Strongly Disagree=1...Strongly Agree=7).

v. **The higher the score, the more empathic the behavioral orientation**.
