# Supplementary material for: Integrating humanities in healthcare: a mixed-methods study for development and testing of a humanities curriculum for front-line health workers in Karachi, Pakistan
Source: Med Humanit. 2024 Jan 17;50(2):372–82. doi: 10.1136/medhum-2022-012576 (PMC11347256; doi:10.1136/medhum-2022-012576)
Supplement: Supplementary data [file medhum-2022-012576supp003.pdf]

Supplementary Material
Process Evaluation Results

Participant Feedback
Across all sessions, on average, participants gave a mean score of 9.7/10 (SD: 0.17) across sessions) when asked to rate the success of the session. The rating was 9.7/10 (SD: 0.16) for the usefulness of the session content and 8.2/10 (SD: 3.3) for the overall satisfaction from the session. LHWs were asked what they liked most about the session and what their key takeaways were, to which the most commonly cited responses could be grouped into themes of 'finding inner peace, hope, self-awareness, self-confidence, and use of songs, role playing and stories as pedagogical tools'. When asked about what they did not like about the session the most commonly cited responses included 'lack of punctuality, lengthy nature of session, and difficultly in understanding poetry'.

Facilitator Feedback

Across all sessions, on average, the facilitator gave the LHWs' participation and responsiveness a rating of 9.4/10 and commented that all LHWs participated actively in the sessions. She also gave positive feedback on the curriculum material and the overall session environment, appreciating the discussion that took place and the relevance of the curriculum to LHWs' work. No changes were recommended by the facilitator to improve the overall sessions. When asked to describe the session, the most common responses by the facilitator were '[we had a] good discussion, [session was] informative, enjoyable and [the class had a] friendly environment'

Observer Feedback

Three key themes emerged from the observers notes. Firstly, that the LHWs were keen to participate in the sessions and also share their personal anecdotes with their fellow LHWs. One of the observers wrote in their notes, "the LHWs said they enjoyed the session and liked listening to others' stories and being able to share their own". Their anecdotes ranged from experiences in the field, personal life, to their perspectives on our culture and their duty as health workers. Many would discuss injustices they had witnessed their clients go through and how they chose to help. The second theme that emerged was that LHWs did not shy away from discussing difficult topics such as adoption, abortion, and domestic violence. Lastly, observers noted that LHWs highlighted the powerful effect the training had on them in terms of self-reflection, and opened up to disclose deeply personal aspects of their lives to elaborate how the training had effected them. As written in the observer notes, one LHW commented, "I loved discussing the things about my past and my own life [in the sessions], before this I had never thought about my own life, but after this training, I've learned to think and have realized how much I have done." Another LHW said, "I had lost everything, I had lost myself, I was broken, and after this training I have felt alive again... I realized that I too am a human who needs to be taken care of, and my family and my siblings have also realized this positive change in me." A LHW also claimed, "My whole world has changed after these training sessions. I believe in myself more. Before, I was unable to speak in front of others in the field, and now I'm not scared anymore."
